# High number of refugees in Italy - which strategy works in Italy?

**DOI:** 10.1192/j.eurpsy.2023.117

**Published:** 2023-07-19

**Authors:** S. Galderisi

**Affiliations:** University of Campania “Luigi Vanvitelli”, Naples, Italy

## Abstract

**Abstract:**

A negative impact on mental health of Ukrainian people who will survive the war is very likely.

Those who leave are exposed to the trauma of leaving behind home, relatives, friends, job, habits, i.e., most of what they had built in their life, and to the unpleasant feeling of knowing nothing of what they will go through.

Mutual support and nurture problem-solving strategies, including favoring family reunion, restoring people dignity and control over the environment, help children recover a more positive social reality, are major protective factors in buffering the impact of war, displacement and related trauma.

By November 2022, Italy had hosted more than 170.000 Ukrainian refugees. The National Service intervention, coordinated by the Civil Protection Department, has developed a Plan for the reception and assistance of the population from Ukraine to uniform the response to the emergency on the national territory. The plan has focused on two aspects: humanitarian assistance and reception. The network for reception is composed of the CAS - Extraordinary Reception Centers and the SAI - Integration Reception System. The Plan also provides measures related to health care and education to ensure mandatory vaccination requirements and the access to the school system for unaccompanied foreign minors. As to plans aimed to address mental health issues, the right to care is guaranteed, but several issues can be identified that limit the impact of the national policies on the needs of this population.

**Disclosure of Interest:**

None Declared

